# Low-molecular-weight lipoprotein (a) and low relative lymphocyte concentration are significant and independent risk factors for coronary heart disease in patients with type 2 diabetes mellitus: Lp(a) phenotype, lymphocyte, and coronary heart disease

**DOI:** 10.1186/1476-511X-12-31

**Published:** 2013-03-07

**Authors:** Tatsuya Suzuki, Shoko Futami-Suda, Yoshimasa Igari, Kentaro Watanabe, Motoshi Ouchi, Kazunari Suzuki, Ken-ichi Sekimizu, Yoshiaki Kigawa, Hiroshi Nakano, Kenzo Oba

**Affiliations:** 1 Department of Internal Medicine (Divisions of Cardiology, Hepatology, Geriatric Medicine, and Integrated Medicine), Nippon Medical School, 1-1-5, Sendagi, Bunkyo-ku, Tokyo, 113-8603, Japan; 2 Hanno Geriatric Hospital, Hanno, Saitama, Japan

**Keywords:** Lipoprotein (a) phenotype, Relative lymphocyte concentration, Coronary heart disease

## Abstract

**Background:**

The aim of the present prospective study was to examine whether lipoprotein (a) [Lp(a)] phenotypes and/or low relative lymphocyte concentration (LRLC) are independently associated with coronary heart disease (CHD) in patients with type 2 diabetes mellitus (T2DM).

**Methods:**

Serum Lp(a) concentration, Lp(a) phenotypes, and RLC were analyzed in 214 subjects. Lp(a) phenotypes were classified into 7 subtypes according to sodium dodecyl sulfate-agarose gel electrophoresis by Western blotting. Subjects were assigned to the low-molecular-weight (LMW (number of KIV repeats: 11–22) ) and high-molecular-weight (HMW( number of KIV repeats: >22 )) Lp(a) groups according to Lp(a) phenotype and to the LRLC (RLC: <20.3%) and normal RLC (NRLC; RLC: ≥20.3%) groups according to RLC. A CHD event was defined as the occurrence of angina pectoris or myocardial infarction during the follow-up period.

**Results:**

During the follow-up period, 30 cases of CHD events were verified. Neutrophil count showed no correlation with CHD, while relative neutrophil concentration and RLC showed positive and negative correlations, respectively, with CHD. The Cox proportional hazard model analysis revealed the following hazard ratios adjusted for LMW Lp(a), LRLC, and LMW Lp(a) + LRLC: (4.31; 95% confidence interval [CI], 1.99-9.32; *P* < 0.01, 3.621; 95% CI, 1.50-8.75; *P* < 0.05, and 7.15; 95% CI, 2.17-23.56; *P* < 0.01, respectively).

**Conclusions:**

Our results suggest that both LMW Lp(a) and LRLC are significant and independent risk factors for CHD and that the combination thereof more strongly predicts CHD in patients with T2DM.

## Background

Recent studies have reported that diabetes mellitus may correlate with coronary heart disease (CHD) [1,2]. However, conventional risk factors for CHD in previous studies do not account for all cases of CHD. Furthermore, epidemiologic studies have consistently shown a significant relationship between white blood cell (WBC) counts and the onset of CHD [3,4]. Similarly to WBC counts, relative lymphocyte concentration (RLC) is also a strong predictor of CHD [5-7]. Inflammatory markers are elevated in acute coronary syndrome (ACS), and ACS is characterized by unstable atherosclerotic plaque that is activated by a systemic inflammatory reaction [8]. Furthermore, low RLC (LRLC) is related to system inflammation [9]. In addition, recent studies have focused on lymphocytes (especially, RLC) and have reported the value of this clinical parameter [10,11].

Lipoprotein (a) [Lp(a)], a plasma complex composed of apolipoprotein (a) [apo(a)] covalently linked to apo B-100 [12], is an important risk factor for CHD [13,14]. Serum Lp(a) is a genetically determined independent risk factor, with a presumed variation range of ≤10% [15]. Furthermore, a recent study has shown that apo(a) has a high degree of genetic polymorphism and that this polymorphism may have a predictive value greater than serum Lp(a) concentration because Lp(a) phenotypes possess a genetic trait not influenced by environmental factors as does serum Lp(a) concentration [15]. However, this factor alone does not fully account for the onset of CHD.

To the best of our knowledge*,* no study in general outpatients with type 2 diabetes mellitus (T2DM) has examined the relationship between apo(a) phenotypes and RLC. Therefore, we examined the effects between each Lp(a) phenotype and RLC on the onset of CHD in patients with T2DM during the follow-up period.

## Methods

### Study participants

The present prospective study enrolled 214 Japanese outpatients with T2DM (115 men and 99 women) who were referred to our hospital since 1995. All subjects provided informed consent. The present study was designed in compliance with the ethic regulations set out by the Helsinki Declaration. They underwent a standardized interview and physical examination. Electrocardiography was performed at baseline. In the present study, CHD was defined as the new onset of angina pectoris or myocardial infarction during the follow-up period. The attending physician and the cardiologist followed the diagnostic criteria to diagnose angina pectoris as an endpoint.

Myocardial infarction was diagnosed by the presence of at least 2 of the following 3 criteria: 1) a clinical history of central chest pressure, pain, or tightness lasting for 30 minutes or longer; 2) an ST segment elevation greater than 0.1 mV in at least 1 standard or 2 precordial leads, an ST segment depression greater than 0.1 mV in at least 2 leads, abnormal Q wave, or T wave inversion in at least 2 leads; and 3) an increase in serum creatine kinase concentration to twofold the upper limit of normal. Angina pectoris was diagnosed based on the repeated episodes of chest pain during an effort that usually disappeared shortly after the cessation of the effort or the sublingual administration of nitroglycerin. Angina pectoris was defined to present an ST segment depression greater than 1 mm on electrocardiograms obtained during chest pain. Patients with T2DM were diagnosed according to the World Health Organi-zation criteria. Patients with hypertension were defined as persons who had a systolic blood pressure (SBP) of 140 mmHg or higher or a diastolic blood pressure (DBP) of 90 mmHg or higher, or who were concurrently receiving antihypertensive drugs. Patients with dyslipidemia were defined as persons who had a serum total cholesterol concentration of 220 mg/dL or higher, a serum low-density lipoprotein cholesterol (LDL-C) concentration of 140 mg/dL or higher, a serum triglyceride concentration of 150 mg/dL or higher, or a serum high-density lipoprotein cholesterol (HDL-C) concentration of 40 mg/dL or lower, or who were concurrently receiving lipid-lowering drugs. Regarding chronic kidney disease (CKD), the estimated glomerular filtration rate was calculated as per the study of Matsuo et al. based on the Japanese Society of Nephrology’s guidelines for CKD, and its grades were determined [16]. HbA1c (%) is given as National Glycohemoglobin Standardization Program (NGSP) equivalent values (%), which were calculated using the following formula [17]: HbA1c (%) = HbA1c (Japan Diabetes Society; %) + 0.4%. LDL-C was calculated using Friedewald’s formula [18].

### Anthropometric measurements

The body mass index was calculated as weight (in kilograms) divided by the square of height (in meters). At screening, subjects who had never smoked and ex-smokers were classified as nonsmokers, while those who were currently smoking were categorized as current smokers.

Exclusion criteria included a history of liver cirrhosis, tuberculosis, patients receiving glucocorticoids over the previous 6 weeks for rheumatoid arthritis, neoplasms, and Basedow disease. To the extent possible, patients with heart failure who had physical activity restrictions (NYHA II or greater) were excluded.

### Assessment of Lp(a) phenotypes and their subgroups

Serum Lp(a) concentrations were determined according to the latex agglutination (LA) method. Within-run CVs ranged from 1.9 to 2.1% and between-run CVs from 2.7 to 3.9% (Lp(a) Latex (Sekisui Medical, Tokyo, Japan). Apo(a) phenotyping was performed according to sodium dodecyl sulfate-agarose gel electrophoresis by Western blotting. Apo(a) phenotypes were classified into 7 subtypes [F, B, S1, S2, S3, S4, and O (null)] [19]. The Lp(a) gene is characterized by extensive size polymorphism caused by a variable number of Kringle IV-2 (KIV-2) repeats that are transcribed and translated into protein isoforms of different sizes [20]. Kronenberg et al. considered 11–22 and >22 KIV repeats as LMW and HMW, respectively. A conventional cutoff margin was established between 640 Kda and 655 Kda to group LMW and HMW apo(a) phenotypes. The LMW group included patients having at least one apo(a) with 11–22 KIV repeats; the HMW group comprised patients having only isoforms with more than 22 KIV repeats [20]. At the commencement of the follow-up period, subjects were assigned to either of the following two groups according to their Lp(a) phenotypes: the LMW (F, B, S1, and S2) Lp(a) group (number of KIV repeats: 11–22) and the HMW (S3, S4, and O) Lp(a) group (number of KIV repeats: >22) as previously described [21,22]. When the subject had a double band, the faster band was used to express the phenotype [23].

### Biochemical measurements

After an overnight fast, blood samples were collected to measure plasma blood glucose levels and serum lipid concentrations. Plasma glucose levels were measured according to the glucose oxidase method. Serum concentrations of total cholesterol, HDL-C, and triglyceride were enzymatically measured with an automatic analyzer. Serum C-reactive protein (CRP) levels were measured by latex agglutination nephelometric immunoassay (LZ test ‘Eiken’ CRP-HG; Eiken Kagaku Co. Ltd, Tokyo, Japan) and with both intra- and interassay coefficients of <1.69%. Blood pressures were measured by the physician who used a standard mercury sphygmomanometer, with the subject in the sitting position after at least a 5-minute rest.

Complete blood counts were measured with an automated cell counter (SE-9000, Sysmex, Corporation, Kobe, Japan) according to the standard techniques*.* Correlations with the manual method for WBC differentials showed good results (neutrophil %, r = 0.942; lymphocyte %, r = 0.937) [24]. The coefficient of variation for repeated measurements of samples from hospitalized patients was maintained at 2.5%. RLC was defined as (total number of lymphocytes/total number of leukocytes) × 100. The normal range of RLC was 20.3 to 50.0%, as defined by the central 95th percentile in a population of healthy subjects [25]. Furthermore, LRLC was defined to present an RLC of <20.3% based on previously reported reference values [26]. Subjects were assigned to two groups according to RLC: the low RLC (LRLC; RLC: < 20.3%) group; and the normal RLC (NRLC; RLC: ≥ 20.3%) group.

### Follow-up

Based on previous studies [10,11,27], patients were followed up for about 7 years as a sufficient follow-up period. Information was also obtained for patients who no longer underwent medical care at the hospital. Follow-up data were obtained by the review of medical records or by the telephone interview. We contacted the attending physician directly after obtaining patient consent to inquire about the presence or absence of any CHD event. When the new attending physician was not known, we contacted the patient by telephone to inquire about his/her current clinical condition. Regarding patients with CHD events, we attempted to directly contact the current attending physician. Information about 50 patients was obtained in the above manner. No information could be obtained for 11 patients; these patients were considered withdrawals from the study. Follow-up was discontinued for 13 patients who died of cancer (n = 6), pneumonia (n = 4), or subarachnoid hemorrhage (n = 3).

### Statistical analyses

The baseline characteristics of subjects in the two study groups were compared by using Student’s *t*-test for continuous variables and the chi-square test for categorical variables. Pearson’s correlation coefficients were used to evaluate correlations between CHD and baseline characteristics. Continuous variables were expressed as mean ± SD. The Cox proportional hazard model was used to investigate the relationship between the onset of CHD and the following explanatory variables. A *P*-value of <0.05 was considered statistically significant. The SPSS software (Statistical Package, version 11.0; SPSS Inc., Chicago, Il, USA) was used for all statistical analyses.

## Results

Baseline characteristics by Lp(a) phenotype of subjects in the HMW Lp(a) group and the LMW Lp(a) group are shown in Table 1. Serum Lp(a) was significantly higher (*P* < 0.001 ) in the LMW Lp(a) group than in the HMW Lp(a) group. Significant differences were not found between the two study groups with regard to other variables. Furthermore, the medication rate of statins/fibrates and the medication rate of ACE-I/ARBs were significantly higher in the LMW Lp(a) group than in the HMW Lp(a) group (*P* = 0.004 and *P* = 0.031, respectively). However, no significant difference was found between the two groups with respect to the medication rates of calcium channel blockers (CCBs), antiplatelets, and the complication rate of peripheral artery disease, stroke, and CKD II/III.

**Table 1:** Baseline characteristics by lipoprotein (a) [Lp(a)] phenotype

**Baseline characteristics**	**All subjects (n = 214)**	**HMW Lp(a) (no. of KIV2 repeats: > 22) group (n = 169)**	**LMW Lp(a) (no. of KIV 2 repeats: 11–22) group (n = 45)**	***P*-value**
Age (years)	62 ± 10	61 ± 10	65 ± 10	0.068
Gender (male/female)	105/99	93/76	22/23	0.489
White blood cells (/mm^3^)	6,197 ± 1563	6,134 ± 1609	6,447 ± 1362	0.250
Neutrophil (/mm^3^)	3,711 ± 1223	3,677 ± 1262	3,838 ± 1057	0.433
Monocyte (/mm^3^)	348 ± 142	360 ± 139	399 ± 152	0.101
Basophil (/mm^3^)	37 ± 46	40 ± 62	34 ± 30	0.527
Eosinophil (/mm^3^)	173 ± 132	170 ± 126	185 ± 154	0.511
Lymphocyte (/mm^3^)	1,889 ± 617	1,866 ± 601	1,973 ± 675	0.305
Neutrophil (%)	59.5	59.5	59.0	0.901
Monocyte (%)	6.0	5.9	6.3	0.353
Basophil (%)	0.6	0.6	0.5	0.302
Eosinophil (%)	2.9	2.9	2.0	0.813
Lymphocyte (%)	31.0	31.0	30.8	0.884
Systolic blood pressure (mmHg)	138 ± 16	138 ± 17	138 ± 15	0.960
Diastolic blood pressure (mmHg)	81 ± 10	81 ± 10	80 ± 10	0.676
Lp(a) (mg/dL)	24.1 ± 26.8	16.2 ± 13.4	53.5 ± 40.9	< 0.001
(median: range)	(14.6: 0–221.0)	(13.0: 0–73.3)	(41.5: 8–221.0)	
Total cholesterol (mg/dL)	211 ± 39	211 ± 40	213 ± 35	0.781
HDL-C (mg/dL)	60 ± 19	61 ± 20	58 ± 17	0.492
Triglyceride (mg/dL)	134 ± 92	134 ± 93	131 ± 92	0.790
LDL-C (mg/dL)	125 ± 31	123 ± 32	129 ± 29	0.270
Creatinine (mg/dL)	1.0 ± 0.4	1.0 ± 0.4	0.9 ± 0.2	0.348
Fasting plasma glucose (mg/dL)	166 ± 65	168 ± 66	162 ± 62	0.503
HbA1c (%)	7.9 ± 1.8	8.0 ± 1.8	7.6 ± 1.6	0.222
Body mass index (kg/m^2^)	23.3 ± 4.1	23.3 ± 4.2	23.6 ± 4.0	0.665
Statins/fibrates (%)	38.8	34.1	57.8	0.004
ACE-I/ARBs (%)	22.0	18.3	35.6	0.031
CCBs (%)	27.6	26.5	33.3	0.364
Antiplatelets (%)	24.3	24.5	23.3	0.862
Peripheral vascular disease	5.1	5.3	4.4	0.819
Stroke (%)	9.8	11.2	4.4	0.091
CKD II/III (%)	93.5	92.9	95.6	0.524
Current smoker (%)	27.5	26.0	32.5	0.412
C-reactive protein (%)^**§**^	86.9	88.1	75.0	0.342

Values are expressed as mean ± SD or numeral (%).

HMW, high-molecular-weight; LMW, low-molecular-weight; §: Proportion of subjects with a C-reactive protein value of < 4.0 mg/L; HDL-C, high-density lipoprotein cholesterol; LDL-C, low-density lipoprotein cholesterol; HbA1c, glycated hemoglobin; ACE-I/ARBs, angiotensin- converting enzyme inhibitors/angiotensin receptor blockers; CCBs, calcium channel blockers; CKD, chronic kidney disease.

Baseline characteristics by RLC are shown in Additional file 1: Table S1. Neutrophil counts and relative neutrophil concentration were significantly higher, and lymphocyte counts and relative monocyte concentration and relative lymphocyte concentration were significantly lower (*P* =0.001, *P* < 0.001, *P* < 0.001, *P* = 0.018, *P* < 0.001, respectively) in the LRLC group than in the NRLC group. Significant differences were not found between the two study groups with regard to other variables. However, no significant difference was found between the two groups with respect to the medication rates of calcium channel blockers (CCBs), antiplatelets, and the complication rate of peripheral artery disease, stroke, and CKD II/III.

Correlation coefficients of baseline characteristics with Lp(a) phenotypes, RLC, and CHD are shown in Table 2. No correlation was found between Lp(a) phenotypes and lymphocyte count and between Lp(a) phenotypes and RLC. Furthermore, negative correlation was found between RLC and neutrophil count and between RLC and relative neutrophil concentration. Neutrophil count showed no correlation with CHD, while relative neutrophil concentration and RLC showed positive and negative correlations, respectively, with CHD. We performed an ROC analysis on CHD with respect to WBC, N (absolute number), Lym (absolute number), % N, and % Lym. The AUC value was slightly greater for % Lym (0.68) than for % N (0.67) and WBC (0.493). In the present study, therefore, we selected % Lym although both had a mirror image. No correlation was found between Lp(a) phenotypes and baseline characteristics except for statins/fibrates and ACE-I/ARBs. Therefore, we regard both Lp(a) phenotypes and RLC as dependent factors.

**Table 2:** Correlation coefficients of lipoprotein (a) [Lp(a)] phenotypes, relative lymphocyt concentration, and coronary heat disease with baseline characteristics during follow-up

**Baseline characteristics**	**Correlation coefficients**		
	Lp(a) phenotypes	RLC	CHD
Age	0.124	- 0.048	0.188**
Female gender	0.050	0.073	0.030
White blood cells (/mm^3^)	0.079	- 0.123	- 0.041
Neutrophil (/mm^3^)	0.054	- 0.380***	0.059
Lymphocyte (/mm^3^)	0.071	0.414***	- 0.184**
Neutrophil (%)	- 0.009	- 0.557***	0.218**
Lymphocyte (%)	- 0.010	0.568***	- 0.215**
Systolic blood pressure	0.004	- 0.014	0.137*
Diastolic blood pressure	- 0.026	- 0.052	0.109
Lp(a)	0.568***	- 0.016	0.318***
Total cholesterol	0.023	0.117	- 0.081
HDL-C	- 0.047	- 0.028	- 0.015
Triglyceride	- 0.015	0.053	- 0.065
LDL-C	0.078	- 0.136*	- 0.077
Creatinine	- 0.064	- 0.008	- 0.038
Fasting plasma glucose	- 0.045	- 0.070	0.080
HbA1c	- 0.084	0.021	0.060
Body mass index	0.031	0.080	0.043
C-reactive protein^**§**^	- 0.072	- 0.113	0.079
Current smoker	0.058	0.078	0.165*
Statins/fibrates	0.201**	- 0.008	0.145*
ACE-I/ARBs	0.165*	0.053	0.108
Antiplatelets	- 0.012	- 0.120	0.184**
CKD II/III	0.044	0.045	- 0.002

Lp(a), lipoprotein (a); HDL-C, high-density lipoprotein cholesterol; LDL-C, low-density lipoprotein.

cholesterol; ACE-I/ARBs, angiotensin-converting enzyme inhibitors/angiotensin receptor blockers; CKD, chronic kidney disease RLC, relative lymphocyte concentration; CHD, coronary heart disease.

*P < 0.05, **P < 0.01, ***P < 0.001.

Table 3 shows the results from an analysis using the Cox proportional hazard model of survival by CHD event during follow-up. Age, female gender, CRP, ACE-I/ARBs, SBP, HDL-C, HbA1c, and CKD II/III were selected as variables to adjust the Cox proportional hazards based on Table 1, Table 3, and Additional file 1: Table S1. The Cox proportional hazard model was used to analyze LMW Lp(a), LRLC, and LMW Lp(a) + LRLC. When not adjusted for age, female gender, CRP, ACE-I/ARBs, SBP, HDL-C, HbA1c, and CKD II/III, hazard ratio for LMW Lp(a), LRLC, and LMW Lp(a) + LRLC were 4.45, 3.45, and 11.31, respectively. When adjusted for these variables, hazard ratio for LMW Lp(a), LRLC, and LMW Lp(a) + LRLC were 4.31; 95% confidence interval [CI], 1.99-9.32; *P* < 0.01, 3.62; 95% CI, 1.50-8.75; *P* < 0.05, and 7.15; 95% CI, 2.17-23.56; *P* < 0.01, respectively.

**Table 3:** Cox proportional hazard model of survival by coronary heat disease event during follow-up

**Variables**	**Hazard ratio**	**(95% CI)**	**Adjusted hazard ratio**	**(95% CI)**
LMW Lp(a) (no. of KIV2 repeats: 11–22)	4.45***	(2.18-9.11)	4.31^†^**	(1.99-9.32)
LRLC	3.45**	(1.48-8.05)	3.62^†^*	(1.50-8.75)
LMW Lp(a) (no. of KIV2 repeats: 11–22) + LRLC	11.31***	(3.81-33.24)	7.15^†^**	(2.17-23.56)

†Adjusted for age, female gender, CRP, ACE-I/ARBs, SBP, HDL-C, HbA1c, and CKD II/III.

CRP, C-reactive protein; ACE-I/ARBs, angiotensin-converting enzyme inhibitors/ angiotensin receptor blockers; SBP, systolic blood pressure; HDL-C, high-density lipoprotein cholesterol; HbA1c, glycated hemoglobin; CKD, chronic kidney disease; LMW Lp(a), low-molecular-weight lipoprotein (a); LRLC, low relative lymphocyte concentration.

*P < 0.05, **P < 0.01, ***P < 0.001.

## Discussion

Our results suggest that not only LMW Lp(a) and LRLC—which are independent predictors for the onset of CHD—but also the combination thereof more strongly predicts CHD. We used the Cox proportional hazard model to analyze LMW Lp(a), LRLC, and LMW Lp(a) + LRLC. When not adjusted, hazard ratio for LMW Lp(a), LRLC, and LMW Lp(a) + LRLC were, 4.45, 3.45, and 11.31 respectively. When adjusted for age, female gender, CRP, ACE-I/ARBs, SBP, HDL-C, HbA1c, and CKD II/III, hazard ratio for LMW Lp(a), LRLC, and LMW Lp(a) + LRLC were, 4.31, 3.62 and 7.15, respectively.

Many previous studies have reported that the incidence of CHD was significantly higher in the elevated Lp(a) group [13,14,28]. Furthermore, Lp(a) has been reported to compete with plasminogen for the binding sites in a dose-dependent manner. In addition, the risk of developing CHD due to Lp(a) was attributed to the amount of Lp(a) that was bound to small-size apo(a) [29]. In the present study, therefore, we investigated how Lp(a) phenotypes, but not serum Lp(a) concentrations, might influence the onset of CHD. We also speculated that every phenotype presents genetic polymorphism as described by Ichinose et al. [30]. Consequently, not only serum Lp(a) concentrations but also Lp(a) phenotypes were found to be associated with the onset of CHD.

We consider that LRLC is a predictor of CHD based on its diagnostic usefulness according to previous studies (5,6,7,11), as well as on the correlation coefficients of CHD (Table 2) and the results from the ROC analysis about blood cell markers in the present study. Data from experimental work in animals and *in vitro* data show that leukocytes also play an important role in atherogenesis [31]. Furthermore, previous investigations have demonstrated that LRLC is also an independent predictor for CHD [5,7]. Our results indicated that subjects with LRLC had an adjusted 3.62-fold hazard ratio of developing CHD events during follow-up and that LRLC was an independent predictor for CHD. Our data support at least partially the role of leukocytes in the chronic process of atherosclerosis. Although pathophysiological mechanisms still remain unclear, current evidence suggests that LRLC may be useful for identifying patients with heart disease, e.g., ACS and heart failure. To the best of our knowledge, however, no previous study has examined whether LMW Lp(a) and LRLC, when combined, are strong and independent predictors for the onset of CHD. Our study is the first to address this issue.

According to many previous studies, the causes of lymphocytopenia are regarded as an early marker of stress because the stress-induced increase in cortisol secretion leads to lymphocytopenia [32,33]. The mechanism to induce changes in leukocyte counts probably involves interactions among the nervous, endocrine, and immune systems that differ from the electrical and cell-damage mechanisms for electrocardiographic and biochemical markers of infarction [6]. The hypothalamus responds by increasing serum corticotropin-releasing hormone levels, which then causes pulsatile increases in serum cortisol levels.

Not only physiological stress but also mental stress has been speculated to provoke the rupture of the fibrous cap around coronary atherosclerotic plaque [34]. Black PH et al. hypothesized that stress induces the release of cytokines, which, together with major stress hormones—corticosteroids and catecholamines, induces the production of acute phase proteins in the liver [35] and that various classes of stress can induce the production and release of proinflammatory cytokines which may mediate micro thrombosis.

There are several limitations to our study. First, we enrolled outpatients in a prospective open-label trial, which may potentially involve attribution biases. Furthermore, our study cannot rule out the presence of selection bias due to its nature of being a hospital cohort study. Second, sample size for the onset of CHD may be small. Therefore, a multicenter study enrolling a greater number of patients will be required in the future. Furthermore, high-sensitivity CRP could not be measured. There is also a need to determine the number of lymphocyte count measurements by multiple measurements in the future. Third, we did not measure serum cortisol levels to confirm its elevation in association with a reduction in RLC. To the extent possible, patients with heart failure (NYHA II or greater) were excluded. However, ejection fraction (EF) was not determined at baseline. Finally, several Lp(a) phenotypes are considered to exist. A further effort should be made to determine whether other unknown Lp(a) phenotypes may or may not correlate with the onset of CHD. Moreover, further study using analytical procedures (e.g., pulse-field electrophoresis of unamplified genomic DNA) will elucidate the effects of Lp(a) with a specified number of KIV-2 repeats on CHD in the future.

## Conclusions

LMW Lp(a) is an independent risk factor for CHD, and LRLC is also an independent risk factor of CHD. LMW Lp(a) and LRLC, when combined, predict CHD more strongly in patients with T2DM.

## Abbreviations

Lp(a): Lipoprotein (a); LMW: Low molecular weight; HMW: High molecular weight; Kringle IV-2: KIV-2; LRLC: Low relative lymphocyte concentration; NRLC: Normal relative lymphocyte concentration; CHD: Coronary heart disease; T2DM: Type 2 diabetes mellitus; LDL-C: Low-density lipoprotein cholesterol; HDL-C: High-density lipoprotein cholesterol; ACE-I/ARBs: Angiotensin-converting enzyme inhibitors/angiotensin receptor blockers; CRP: C-reactive protein; CKD: Chronic kidney disease; SBP: Systolic blood pressure

## Competing interests

The authors declare that they have no competing interests.

## Authors’ contributions

TS and KO contributed to the study conception and design, and writing manuscript. YI, KW and SFS participated in data collection, data analysis. MO, KS and KS contributed to design, conduct, data collection. YK, HN contributed to design, conduct and analysis. All authors read and approved the final manuscript.

## Supplementary Material

Additional file 1: Table S1 Baseline characteristics by relative lymphocyte concentration. Click here for file
