# Supplementary material for: Low-molecular-weight lipoprotein (a) and low relative lymphocyte concentration are significant and independent risk factors for coronary heart disease in patients with type 2 diabetes mellitus: Lp(a) phenotype, lymphocyte, and coronary heart disease
Source: Lipids Health Dis. 2013 Mar 7;12:31. doi: 10.1186/1476-511X-12-31 (PMC3606419; doi:10.1186/1476-511X-12-31)
Supplement: Additional file 1: Table S1 — Baseline characteristics by relative lymphocyte concentration. [file 1476-511X-12-31-S1.doc]

Supplement 1 Baseline characteristics by relative lymphocyte concentration

| Baseline characteristics | All subjects  (n = 214) | NRLC group  (n = 194) | LRLC group  (n = 20) | *P*-value |
| --- | --- | --- | --- | --- |
| Age (years) | 62 ± 10 | 62 ± 10 | 64 ± 10 | 0.484 |
| Gender (male/female) | 105/99 | 103/92 | 22/23 | 0.292 |
| White blood cells (/mm^3^) | 6,197± 1563 | 6,136 ± 1493 | 6,795 ± 2083 | 0.182 |
| Neutrophil (/mm^3^) | 3,711 ± 1223 | 3,562 ± 1062 | 5,152 ± 1696 | 0.001 |
| Monocyte (/mm^3^) | 348 ± 142 | 371 ± 142 | 337 ± 143 | 0.312 |
| Basophil (/mm^3^) | 37 ± 46 | 40 ± 59 | 27 ± 21 | 0.315 |
| Eosinophil (/mm^3^) | 173 ± 132 | 173 ± 134 | 170 ± 118 | 0.922 |
| Lymphocyte (/mm^3^) | 1,889 ± 617 | 1,971 ± 579 | 1,097 ± 375 | < 0.001 |
| Neutrophil (%) | 59.5 | 57.8 | 75.5 | < 0.001 |
| Monocyte (%) | 6.0 | 6.1 | 5.0 | 0.018 |
| Basophil (%) | 0.6 | 0.6 | 0.4 | 0.174 |
| Eosinophil (%) | 2.9 | 2.9 | 2.7 | 0.714 |
| Lymphocyte (%) | 31.0 | 32.5 | 16.2 | < 0.001 |
| Systolic blood pressure (mmHg) | 138 ± 16 | 138 ± 17 | 139 ± 14 | 0.834 |
| Diastolic blood pressure (mmHg) | 81 ± 10 | 81 ± 10 | 82 ± 7 | 0.446 |
| Lp(a) (mg/dL) | 24.1 ± 26.8 | 23.9 ± 27.4 | 25.5 ± 20.8 | 0.814 |
| (median: range) | (14.6: 0-221.0) | (14.4: 0-221.0) | (21.0: 1-64.5) |  |
| Total cholesterol (mg/dL) | 211 ± 39 | 212 ± 38 | 197 ± 45 | 0.087 |
| HDL-C (mg/dL) | 60 ± 19 | 60 ± 19 | 62 ± 25 | 0.683 |
| Triglyceride (mg/dL) | 134 ± 92 | 135 ± 95 | 118 ± 63 | 0.443 |
| LDL-C (mg/dL) | 125 ± 31 | 126 ± 31 | 111 ± 34 | 0.077 |
| Creatinine (mg/dL) | 1.0 ± 0.4 | 1.0 ± 0.4 | 1.0 ± 0.2 | 0.909 |
| Fasting plasma glucose (mg/dL) | 166 ± 65 | 165 ± 66 | 180 ± 55 | 0.312 |
| HbA1c (%) | 7.9±1.8 | 8.0±1.8 | 7.6±1.9 | 0.758 |
| Body mass index (kg/m^2^) | 23.3 ± 4.1 | 23.4 ± 4.2 | 22.2 ± 2.9 | 0.269 |
| Statins/fibrates (%) | 38.8 | 38.7 | 40.0 | 0.907 |
| ACE-I/ARBs (%) | 22.0 | 22.7 | 15.0 | 0.444 |
| CCBs (%) | 27.6 | 27.8 | 25.0 | 0.788 |
| Antiplatelets (%) | 24.3 | 22.6 | 40.0 | 0.149 |
| Peripheral vascular disease | 5.1 | 4.6 | 10.0 | 0.455 |
| Stroke (%) | 9.8 | 8.8 | 20.0 | 0.245 |
| CKD II/III (%) | 93.5 | 93.8 | 90.0 | 0.514 |
| Current smoker (%) | 27.5 | 28.6 | 16.7 | 0.231 |
| C-reactive protein (%)^§^ | 86.9 | 88.2 | 82.2 | 0.212 |

Values are expressed as mean ± SD or numeral (%).

HMW, high-molecular-weight; LMW, low-molecular-weight; §: Proportion of subjects with a CRP value of

< 4.0 mg/L; HDL-C, high-density lipoprotein cholesterol; LDL-C, low-density lipoprotein cholesterol,

ACE-I/ARBs, angiotensin-converting enzyme inhibitors/angiotensin receptor blockers; CCBs, calcium channel blockers; CKD, chronic kidney disease; NRLC, normal relative lymphocyte concentration; LRLC, low relative lymphocyte concentration
